# Myosin-1 inhibition by PClP affects membrane shape, cortical actin distribution and lipid droplet dynamics in early Zebrafish embryos

**DOI:** 10.1371/journal.pone.0180301

**Published:** 2017-07-05

**Authors:** Prabuddha Gupta, René Martin, Hans-Joachim Knölker, Deepak Nihalani, Deepak Kumar Sinha

**Affiliations:** 1 Dept. of Biol. Chem., IACS, Kolkata, India; 2 Department Chemie, TU Dresden, Dresden, Germany; 3 Dept. Medicine, Medical University of South Carolina, Charleston, South Carolina, United States of America; Semmelweis Egyetem, HUNGARY

## Abstract

Myosin-1 (Myo1) represents a mechanical link between the membrane and actin-cytoskeleton in animal cells. We have studied the effect of Myo1 inhibitor PClP in 1–8 cell Zebrafish embryos. Our results indicate a unique involvement of Myo1 in early development of Zebrafish embryos. Inhibition of Myo1 (by PClP) and Myo2 (by Blebbistatin) lead to arrest in cell division. While Myo1 isoforms appears to be important for both the formation and the maintenance of cleavage furrows, Myo2 is required only for the formation of furrows. We found that the blastodisc of the embryo, which contains a thick actin cortex (~13 μm), is loaded with cortical Myo1. Myo1 appears to be crucial for maintaining the blastodisc morphology and the actin cortex thickness. In addition to cell division and furrow formation, inhibition of Myo1 has a drastic effect on the dynamics and distribution of lipid droplets (LDs) in the blastodisc near the cleavage furrow. All these results above are effects of Myo1 inhibition exclusively; Myo2 inhibition by blebbistatin does not show such phenotypes. Therefore, our results demonstrate a potential role for Myo1 in the maintenance and formation of furrow, blastodisc morphology, cell-division and LD organization within the blastodisc during early embryogenesis.

## Introduction

**Fig 1:**
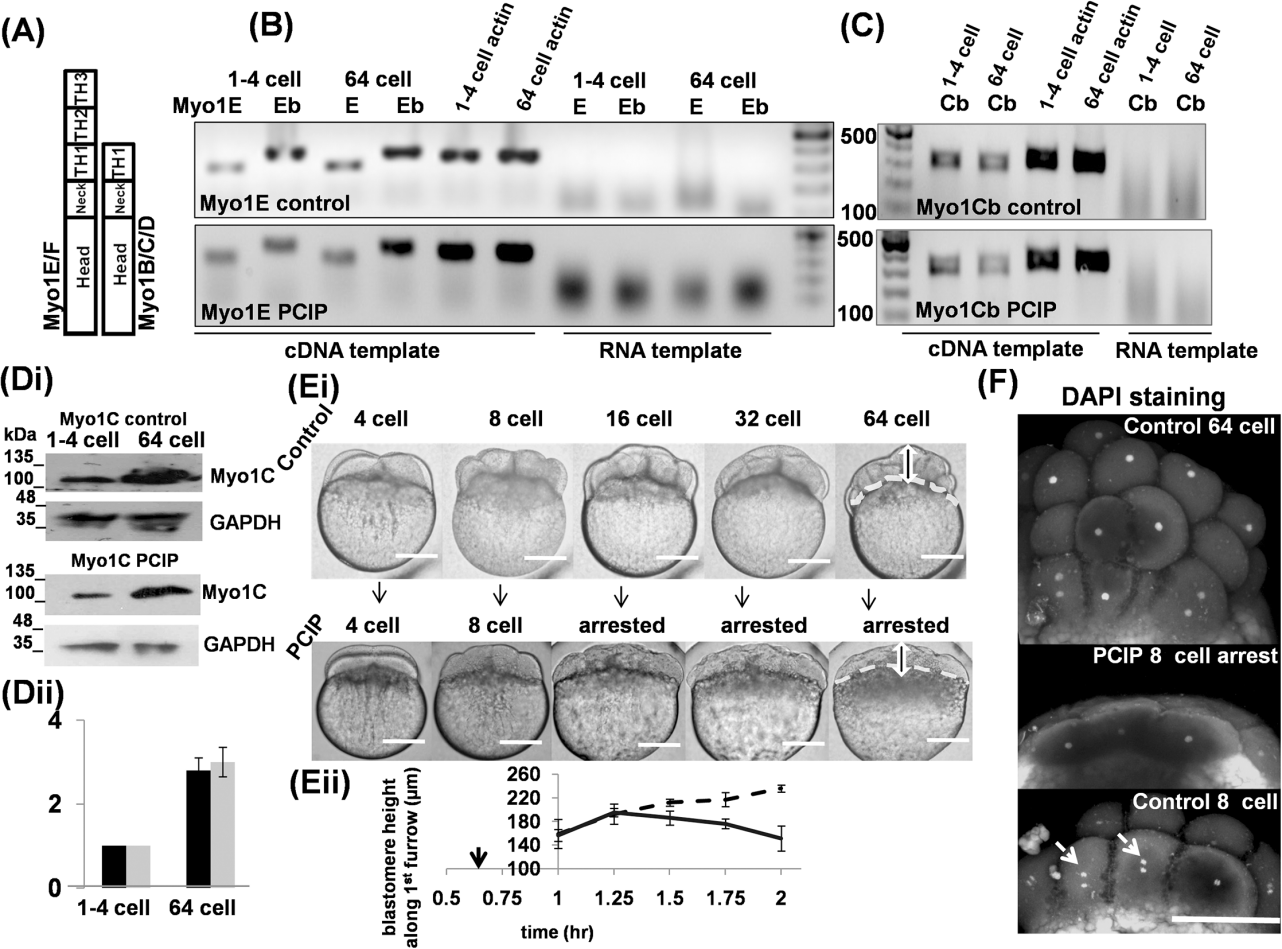
Inhibition of Myo1 arrests cell division and affected blastomere shapeof Zebrafish embryos. Schematic representation of Myo1 domain structures. (A) Semiquantitative RT-PCR profiles from cDNA and RNA templates. For Myo1Ea&b, control (top panel) and with PClP (bottom panel), were compared at 1–4 and 64 cells stages for cDNA and RNA templates, (B) Semiquantitative RT-PCR profiles from cDNA and RNA templates. For Myo1Cb, control (top panel) and with PClP (bottom panel), were compared at 1–4 and 64 cells for cDNA and RNA templates. (C) (i) Western blot for Myo1C relative-levels at 1–4 and 64 cell stages. Control (top panel) and with PClP (bottom panel), GAPDH used as loading control. (ii) Relative change in levels of Myo1C ±PClP, 1–4 and 64 cell stages, control grey, PClP black. Error bars indicate SD, n = 3. (D) (i) Top panel, control embryos at different developmental stages. Bottom panel, PClP treated embryos taken in identical time as in control, dotted line shows boundary between yolk and blastodisc in both panels, (ii) measurement of changes in blastodisk thickness, as indicated by vertical both sided arrows in(Ei) with time, approximately along first cleavage furrow in both, the control (dotted line) and PClP treated embryos (solid line), n = 8, error indicate SD. Black arrow-head in time axis indicates PClP addition. Embryos were observed in lateral or side view position. (E) DAPI stained nucleus profile of 64-cell control (2 hpf) (top panel) and equivalent 2 hpf PClP inhibited 8- cell embryo (middle panel). 1 hpf control 8- cell embryo, showing divided nucleus is also shown (lower panel). bar 120μm in all places.

Myo1 proteins are ATP-driven actin-bound motor proteins that are commonly monomeric (single headed) in nature, unlike dimeric Myosin II (Myo2) molecules [1]. Myo1 isoforms associate with cell membrane by the Tail Homology 1 (TH1) domain that contains a lipid-binding, PH like domain [2, 3]. Myo1 proteins are further classified as short tailed (eg 1B/1C/1D) or long tailed (eg 1E/1F) based on the absence or presence of glycine/proline/alanine rich (TH2) and SH3 domains (TH3) in the tail region (Fig 1A) [4]. Collectively, Myo1 isoforms help in mechanical regulation of membrane architecture by coupling it with actin cytoskeleton [4, 5]. Various Myo1 isoforms have specialized functions [6, 7]. The common theme behind Myo1 function is that they are activated in the presence of F-actin, near membranous structures, eg in membrane-cytoskelatal adhesion, during microvilli vesicle shedding, endo-exocytosis, lipid raft transport and sensory channel gating/adaptation [4, 8].

Zebrafish embryos have thick cortical actin band, in the early stages of development (1–4 cell) [9–11]. They also contain numerous dynamic lipid droplets (LDs) in the cortical region of the blastodisc [11]. These LDs have a neutral lipid-sterol ester core surrounded by a phospholipid monolayer [12]. Recruitment of LDs to the blastodisc of Zebrafish embryos is a cytoskeletal actin dependent process [11]. As Myo1 can bind to both, the actin-cytoskeleton and the lipid membrane, it might mechanically link the cortical actin to plasma membrane in Zebrafish embryos. Similarly, Myo1 could be an important player in regulating the dynamics of LDs, since the dynamics are controlled by actin-cytoskeleton-remodeling in Zebrafish embryos [11]. Membrane-cortical actin linkage is also a key regulator of cell morphogenesis [13]. Since Myo1 is a regulator of membrane cytoskeletal adhesion, we postulate that Myo1 may be critical for the maintenance of blastomeric shape in Zebrafish embryosas well.

We used the drug PClP (pentachloropseudilin) to inhibit themotor activity of Myo1 molecules specifically and examined associated phenotypic changes during early (first to third) cell divisions of Zebrafish embryos [14, 15]. PClP has been used in the past to inhibit motor activity of Myo1 in living cells [15–19]. IC_50_ values for various Myo1 isoforms of different organisms range from 1 to5.6 μM of PClP, whereas for Myo2, Myo5, Myo6 and -Myo7 it is above 90μM [15]. Therefore, at a concentration below 5.6 μM, PClP is a specific inhibitor of Myo1 function. Since Myo1 family is composed of various genes (a, b, c, d, e, f, g, h, that encode for different isoforms [4]), addition of an appropriate concentration of PClP is a more effective way to investigate the overall Myo1 function than generating simultaneous knockout of all isoforms in multiple loci at organism level. We envisioned a global inhibition of all Myo1 isoforms, without affecting other Myosins. It is reported that at 1 μM concentration PClP specifically blocks Myo1C *in vivo* [8, 15]. Therefore, we have chosen to use a concentration of 2.5 μM of PClP throughout this study, which is predicted to inhibit all Myo1 isoforms without affecting non-Myo1 Myosins [8, 15–19].

Myo1 genes are duplicated in Zebrafish, following the general trend of fish genome [20]. Expression profiles of duplicated Zebrafish Myo1Ea&b, Myo1Ca&b, Myo1B and Myo1F has been published and are available in ZFIN database (Thisse *et al*., 2004, direct submission) [21–23]. Among them, Myo1F is associated with hematopoietic cells and it may not be present during early (1–4 cell) embryonic division [21, 24]. Up to 64-cell stage, Myo1F transcript levels are 20–30 fold less than Myo1E [25]. Similarly, Zebrafish Myo1Ca contains the sequence “GRRKAKHRRWAAD” which resembles the published nuclear localization signal of human nuclear Myo1C “GRRKAAKRKWAAQ” [26]. The nuclear effect of Myo1 will not be discussed, since transcription and other chromatin related activities do not take place at 1–8 cell Zebrafish embryos [27–31]. Taken together, we have chosen Zebrafish Myo1Ea/b and Myo1Cb as representative candidates for long and short tailed Myo1 respectively for this study.

The role of Myo1 in early development of the Zebrafish embryos has not been explored previously. Herein, we investigated the role of Myo1 in maintaining the thick cortical actin cortex and the morphology of blastodisc. Additionally, we investigated if Myo1 is critical for LDs recruitment and transportation. We described changes in actin cortex, morphology of blastodisc and LD motion upon Myo1 inhibition by PClP, which provides a basis for studies on the role of Myo1 in early embryonic maturation.

## Result and discussion

### Inhibition of Myo1 by PClP prevents division of blastomeric cells in early Zebrafish embryos

PClP is known to inhibit the functions of all isoforms of Myo1*in vitro* [15] and *in vivo* [16–19]. First we investigated whether PClP will also affect Myo1 concentration *in vivo*, in Zebrafish embryos. We added 2.5 μM PClP (as discussed above) to the embryos within 30–40 minutes post fertilization (mpf) throughout this study (Panel A in S1 Fig). We checked semi-quantitative RT-PCR profile of candidates from short and long-tailed Myo1, Myo1Cb &1E respectively, in control embryos at 40 mpf, (1–4 cells stage) and 2 hpf (64 cells stage). Both Myo1E and 1Cb bands were detected at these time points (Fig 1B and 1C, top panels).

The RT-PCR profiles of PClP treated embryos were compared with control embryos from an identical batch, processed at the same time. The Myo1E and 1Cb were also detected at both the time-points in PClP treated embryo and qualitatively no significant change was observed when compared to the control (Fig 1B and 1C, bottom panels, respectively). Identical observation was made in Myo1B RT-PCR profile (Panel D in S1 Fig).

Furthermore, we checked the relative concentration change of Myo1C protein by western blot. Myo1C was chosen as a candidate because of its established role in lipid transport and cortical granule compensatory endocytosis, along with its role in membrane cytoskeleton adhesion [6, 32]. The antibody used recognizes both Myo1Ca&b [22]. We found that the Myo1C concentration increases approximately by 2.8± 0.3 fold in 64 cells stage compared to 1–4 cells stage (Fig 1Di top panel and Fig 1Dii), in control embryos. This concentration change is in agreement with relative mRNA levels of this protein in these two stages, as reported previously [25]. PClP treated embryos, originating from the same pair of fish and collected at same time-points as above, also showed 2.9±0.35 fold increase of Myo1C (Fig 1Di bottom panel and Fig 1Dii). These results suggest the effect of PClP on embryonic cell division is due to a functional inhibition of Myo1 activity and may not be due to changes of levels inMyo1 expression.

To observe the gross phenotype of Myo1 inhibition, the embryos were immobilized with 0.8% low melting point (LMP) agarose and treated with either the carrier DMSO (control) or with PClP diluted in E3 media. The control embryos were imaged every 15–20 min, as they divided from 4-cell to 64-cell stages (Fig 1Ei top panel). Similarly, we imaged PClP treated embryos at the same frequencyand found a cell division arrest at 8-cell stage (Fig 1Ei bottom panel). The comparison of embryonic morphology of control embryos with PClP treated embryos revealed that cell division apparently proceeded normally when treated with PClP, up to 8-cell stage (Fig 1Ei bottom panel). Additionally, when the control embryos developed to multilayer 32–64 cells, the PClP treated embryo became a syncytium with no cell septas, probably due to a gradual septal dissolution (compare Fig 1Ei top and bottom panels, dissolved septas could be seen in inhibited embryos after 8-cell time point, side view). Cell division arrest at 8 cell stage followed by septal dissolution could also be observed from top-view position (Panel B S1 Fig -control and panel C in S1 Fig Myo1 inhibited). The absence of clearly demarcated septa on observation with a bright-field optical microscope was used as a working definition of septal dissolution throughout this study. It should be mentioned that our experiments cannot rule out the possibility of the existence of furrows that are not visible in optical imaging.

The PClP treated embryos showed a gradual shrinkage of blastomere thickness, compared to control embryos (Fig 1Ei and 1Eii). Up to 4-cell stage, we did not observe any significant difference in the blastodisc thickness between control and PClP treated embryos (159±24 μm and 156±10 μm respectively, Fig 1Eii). While in control embryos, the thickness of blastodisc gradually increased to 236±6 μm by 64 cell stage, in PClP treated embryos, after a brief rise up to 8 cell stage (195±7 μm) the blastodisc gradually shrank to 151±21 μm (Fig 1Ei and 1Eii). This observation is in agreement with our hypothesis that Myo1 molecules might have a role in blastodisc-morphogenesis.

We further investigated if PClP mediated arrest in cytokinesis was accompanied by an arrest in the nuclear division. We observed that, under Myo1 inhibited conditions, majority of the embryos got arrested at 8 cell stage (2 hrs post fertilization), whereas the control embryo reached 64 cell stage (Fig 1F, middle and top panels respectively). While, 8-cell control embryos had 16 nuclei, the PClP treated embryo has only 8 nuclei. The control 8 cell embryo contains 16 nuclei probably due to the delay between anaphase and cytokinesis (arrows, Fig 1F, bottom panel) [33].

Nuclear variants of Myo1 isoforms are associated with transcription and chromatin remodeling, both of which are silent during early cell divisions of Zebrafish embryos [27–31]. In the literature, there are no reports on interaction of Myo1 with DNA polymerase. DNA repair checkpoints are also non-operational in the first few divisions of Zebrafish embryos [31]. Therefore, nuclear division arrest by PClP might not be due to a direct inhibition of DNA replication. Thus, we speculate that there might be non conventional checkpoints in embryos, which prevent nuclear division in the embryos, if function of any major cytoskeletal protein is compromised.

In conclusion, we found that Myo1-inhibition by PClP, arrests the karyokinesis and cytokinesis in Zebrafish embryos. Myo2 inhibition also leads to cell division arrest and dissolution of cleavage furrows [34]. Therefore, we examined similarities and differences in the cell division arrest and changes in cell septa in blastomere upon Myo1 and Myo2 inhibition. We speculate that Myo1 molecules might be active in the furrow, as it was rich in newly polymerized actin and close to membrane surface from either of the two daughter cells [35].

### Furrow formation phenotype mediated by inhibition of Myo1 differs from that of Myo2

Inhibition of Myo2 by blebbistatin inhibits cell division of Zebrafish embryos [34, 36]. We compared the furrow formation defects as a result of Myo1-inhibition (PClP) with that of Myo2 (Blebbistatin). As reported [34], we observed blebbistatin treatment (at 10–15 mpf) leads to cleavage furrow ingression, however the furrow fails to mature, resulting in regression (S2 Fig). As PClP addition at 10–15 mpf permanently freezed the blastomere of embryos at one cell stage, we excluded this time point from the present study. Whereas, PClP addition at 30–40 mpf, when the first cleavage furrow was forming (Panel A in S1 Fig), led to cell division arrest at 8 cell stage (Fig 1E and Fig 1F, panel F in S1 Fig) We then investigated what would have happened if blebbistatin was independently added at the same time-point when PClP was added (during the initiation of first cleavage furrow, panel A in S1 Fig).

**Fig 2:**
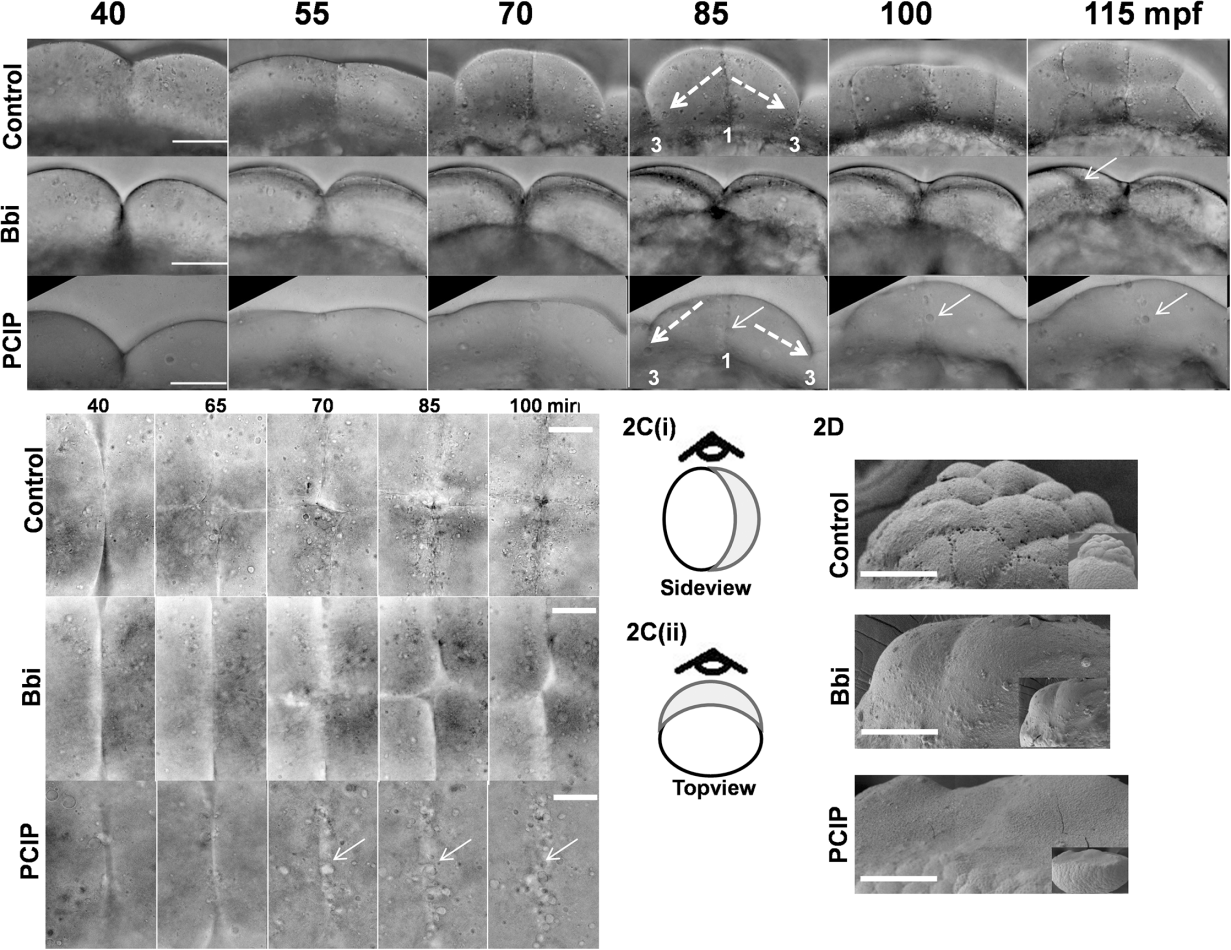
Comparison between role of Myo2 and Myo1, in furrow formation and LD dynamics. (A) Sideview, time-lapse profile of furrow maturation in control (top panel), Blebbistatin treated/Myo2 inhibited (middle panel) and PClP treated/Myo1 inhibited embryos (bottom panel). White dashed arrows in top panel and bottom panel indicate the formation of third furrow (marked as 3), parallel to the first furrow (marked as 1). Filled white arrows in bottom panel indicate the dissolving first-furrow and LD accumulation at that site, in Myo1 inhibited embryo. Filled white arrow in middle panel indicates dis-localization of second and third furrow. Bar 100 μm. (B) Top-view time-lapse profile of furrow maturation in control (top panel), Myo2 inhibited (middle panel) and Myo1 inhibited embryos (bottom panel). Arrows in bottom panel indicate LD accumulation along dissolving first furrow in Myo1 inhibited embryo. Bar 50 μm. (C) Cartoon diagram of embryo with (i) top and (ii) sideview position shown by eye symbol. (D) Scanning electron microscopy showing embryo blastomere surface of 64 cell control (top panel), equivalent time Myo2 inhibited (middle panel) and equivalent time Myo1 inhibited embryo (bottom panel). Bar 100 μM.

We compared control, Myo2 inhibited and Myo1 inhibited embryos in side-view and top-views (Fig 2A top, middle and bottom panels respectively, Fig 2Ci, schematic, Fig 2B). We started our observations from about 40 mpf, when the first furrow had formed (leftmost panels in Fig 2A, Fig 2B and panel A in S1 Fig). While the PClP treated embryo progressed upto 8 cell stage with formation of proper furrows, the blebbistatin treated embryos failed to form any new mature furrows (Fig 2A-lower and middle panels respectively, panels F and G in S1 Fig, S1 Movie). In PClP treated embryos, the 8-cell blastomere at 80–85 mpf subsequently lost all the furrows, forming a flattened syncytium like structure (Fig 2A, bottom panel & panel F in S1 Fig, a longer time observation). Unlike PClP treatment, in case of Myo2 inhibition, the first cleavage furrow never dissolved till 160 mpf (Fig 2A, panel G in S1 Fig, S1 Movie). Also, no subsequent furrows went for full ingression or maturation (Fig 2A, panel G in S1 Fig and S1 Movie). From the top view, a similar observation was made for control and drug treated (blebbistatin and PClP) embryos (Fig 2B, S2 Movie). Taken together, our observations indicate difference in cleavage furrow stability upon Myo1 and Myo2 inhibition.

It has been noted recently that Zebrafish blastomere contains dynamic lipid droplets (LDs) in the cortical region ([11], S1 and S2 Movies). In the control and Myo2 inhibited embryos, we could detect such dynamic LDs on either side of the first cleavage furrow line (Fig 2B top and middle panels respectively, S2 Movie). In Myo1 inhibited embryos, gradual accumulations of LDs were seen on the furrow line itself (Fig 2B, bottom panel-and Fig 2A bottom panel, filled arrows, S2 Movie). Therefore, the absence of LD accumulation at the furrow line in control and Myo2 inhibited embryos indicate that LD dynamics near cleavage furrow might have affected differently by inhibition of Myo1 and Myo2 (Fig 2B top and middle panels, S2 Movie).

To investigate changes in surface profile of embryos in greater detail, we imaged the surface profile of, control, Myo2 inhibited and Myo1 inhibited embryos at 2 hpf by scanning electron microscopy (Fig 2D top, middle and bottom panels). In control, we found a honeycomb like blastomere of 64+ cells (Fig 2D top panel). For Myo2-inhibited embryos, we could see one cleavage furrow in otherwise smooth cell surface (Fig 2D middle panel). In case of Myo1-inhibited embryo, the blastomere surface appeared flatter, uneven and partly sunk in the yolk, in agreement with images above (Fig 2D bottom panel, Fig 1E and 1F, panel F in S1 Fig). Unlike control and Myo2-inhibited embryos, we could not detect any furrow (cell-cell boundary) in 2 hr PClP treated embryos (Fig 2D bottom panel). This observation reconfirms the role of Myo1 in maintaining the pre formed furrow. The unique uneven nature of the surface of Myo1 inhibited embryo could be the result of changes in membrane cytoskeletal interaction, due to the lack of Myo1 mediated link between cytoskeleton and membrane [4, 5].

Taken together, we have found major qualitative differences between the effects of Myo1- and Myo2- inhibition in early blastulation of Zebrafish embryos. Due to structural difference in the active site, IC_50_ values for Blebbistatin to inhibit Myo1 is 50–100 fold high compared to Myo2 [37]. Therefore, we did not attempt experiments with higher concentration of blebbistain. Even if observations related to Myo1 inhibitions could be photocopied at very higher dosage of blebbistation, the concentration of the drug would be much higher than physiologically relevant range. Myo2 activity is required for formation of cleavage furrow and has minimal effect in maintenance of preformed furrows during blastulation. On the other hand, Myo1 is critical for both formation and maintenance of cleavage furrow during blastulation. While Myo2- inhibition had no significant effect on the distribution of LDs in the cortical blastomere, Myo1- inhibition affected the distribution of LD significantly, causing a gradual accumulation of LD at the first furrow line (Fig 2B bottom panel). The surface of Myo1- inhibited embryo appeared rough (Fig 2D bottom panel), which might be due to changes in membrane-actin cytoseleletal interactions. Therefore, we further investigated Myo1-inhibition induced changes in the cortical-actin structure.

### Myo1 maintains morphology of cortical actin and blastomeric membrane

**Fig 3:**
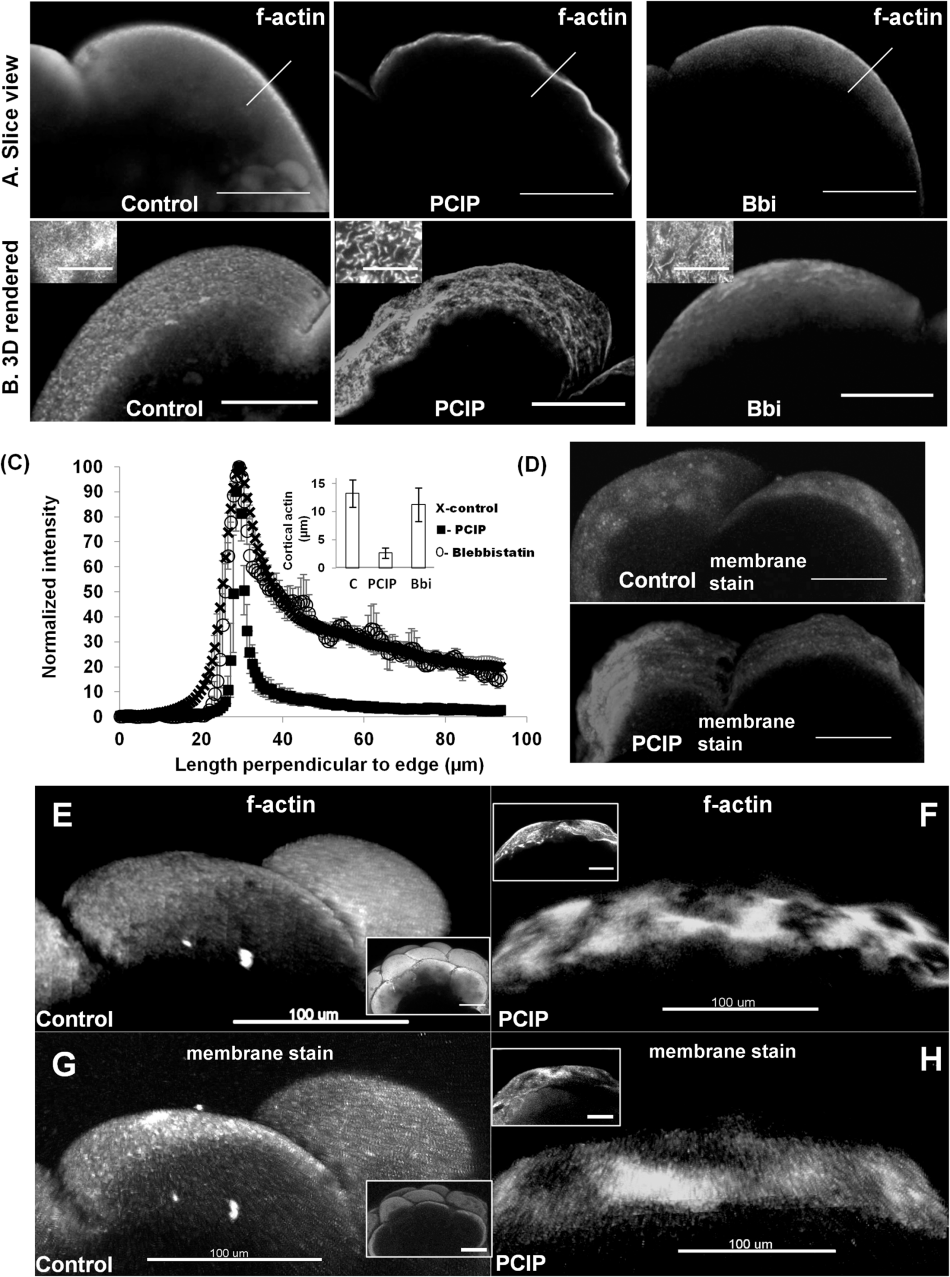
Reorganization of blastomeric cortical actin and membrane upon MyoI inhibition by PClP. (A) Slice views- Cortical actin distribution profile in control (carrier DMSO treated) 1hpf (left panel), PClP treated 1 hpf (middle panel) and blebbistatin treated 1 hpf embryo (right panel). (B) 3D rendered in 100 μm cross section of embryos- Cortical actin distribution profile in control 1hpf (left panel), PClP treated 1 hpf (middle panel) and blebbistatin treated 1 hpf embryos (right panel). Insets, zoomed in view by TIRF imaging. (C) Normalized actin (phalloidin-488) intensity profile perpendicular (along arrows in (A)), to the surface in control (cross), PClP (box) and blebbistatin (circle) treated embryos. Inset- comparison of cortical actin thickness measured by FWHM (n = 5 embryo each, error bars indicate standard deviation), along the line as indicated in Fig 3A. (D) 3D rendered in 100 μm cross section of embryos- Menbrane surface is stained with cell mask plasma membrane dye, control 1 hpf (top panel) and PClP treated 1hpf (bottom panel). (E-H) 3D rendered in 100 μm cross section of embryos. (E) Cortical actin distribution by phalloidin-alexa-488 staining in control embryo, 2 hpf. Inset- full blastomere. (F) Cortical actin distribution in 2 hpf Myo1 inhibited embryo by PClP, phalloidin-alexa-488 staining. Inset- full blastomere. (G) Membrane surface is stained with cell mask plasma membrane dye in control 2 hpf embryo. Inset- full blastomere. (H) Membrane surface is stained with cell mask plasma membrane dye in 2 hpf Myo1 inhibited embryo by PClP. Inset- full blastomere. Scale bars are 100μm everywhere in this figure except 5 μm in zoomed inset of (A&B).

Zebrafish embryo cells are much larger in size compared to average somatic cells (blastomere radius ranging ~200μm) [38]. Maintenance of huge blastomeric cell-size and high mechanical activity due to a short cell-cycle time (20 min) requires a thick cortical actin compared to somatic cells (<1 μm in somatic cells, upto 15 μm in embryos) [35, 39–41]. Myo1 molecules are one of the major regulators of membrane actin-cytosketelon interaction [4, 5]. Therefore, it is likely that there would be discernible changes in the cortical actin and/or membrane profile in Zebrafish embryos, if Myo1 is inhibited. Indeed, compared to control, unique changes were observed in cortical actin distribution, as determined by the phalloidin-488 staining of four cell (1-hpf) embryos treated with 2.5 μM PClP or 100 μM blebbistatin (control-Fig 3A and 3B left panels, PClP-Fig 3A and 3B middle panels & blebbistatin-Fig 3A and 3B right panels respectively, Fig 3C and S3 Movie).

We found that the cortical actin was constricted and became 3–4 fold narrower and brighter than control in Myo1 inhibited embryo (as measured by full width at half maxima (FWHM, Fig 3A and 3B- left and middle panels, Fig 3C & S3 Movie). FWHM changed from 13.26±2.45 μm to 3±1.2 μm upon Myo1 inhibition. No such change in FWHM was observed in case of Myo2 inhibited embryos (Fig 3A and Fig 3B-right panels, Fig 3C & S3 Movie). Overall, 3D rendered cortical actin shape in control and Myo2 inhibited embryos appeared much smoother than in the Myo1 inhibited embryo, which in turn were wrinkled (compare Fig 3B middle panel with Fig 3B left and right panels). This suggests that Myo2 and Myo1 have different roles in maintaining the integrity of actin cytoskeleton. While Myo1 maintains the consistency of cortical actin, Myo2 seems to play no such role. The membrane surface also appeared wrinkled in Myo1 inhibited embryos, compared to the control (as observed after staining with a membrane dye) (Fig 3D).

To further investigate changes in the actin structure, we imaged the Zebrafish cortical actin upon inhibition of Myo1 and Myo2 by 100x TIRF microscopy (Fig 3B insets, S3 Fig). It has been shown that depletion of Myo1C results in loss of filamentous actin, leading to excess actin foci and stress fibers in Huh-7 cells [42]. However during Myo2 inhibition, stress fibers are dissolved and filament like actin structures are favored [43]. We observed that in control and Myo2 inhibited embryos, the surface actin profiles resembled mesh-like structure of thin filaments. Although the Myo2 inhibition resulted in few thicker actin filaments in the mesh structure than control, it had no effect on the thickness of cortical actin-layer as discussed previously (Fig 3B left and right panel insets respectively, S3 Fig). In case of Myo1 inhibited embryos, the cortical actin-layer became thinner and the entire actin filaments became thicker and tubule like (Fig 3B middle panel inset, S3 Fig). This change in cortical actin structure might be a result of functional inhibition of Myo1 isoforms, which stabilizes short filamentous actin [42]. Thus, it is certain that, unlike Myo2, Myo1 inhibition changed both texture and thickness of cortical actin. In the current study, changes in the finer biophysical properties of actin cortex (eg, measurement of changes in cortical tension) were not investigated.

We then inspected the two hpf control (Fig 3E and 3G) and Myo1 inhibited (Fig 3F and 3H) embryos. Visual observation of these embryos suggested presence of a wrinkled surface upon Myo1 inhibition, in agreement with what was shown above (Fig 1E, panel F in S1 Fig). Those wrinkles were exclusively present in both the cortical actin and membrane of Myo1 inhibited embryos and not in control embryos (Fig 3E and 3F cortical actin, Fig 3G and 3H membrane surface). This observation suggests that Myo1 is necessary for cortical actin-membrane stability during the entire duration of early embryogenesis (0–2 hpf). We further investigated how the loss of Myo1 mechanical-link between actin and membrane could generate wrinkles in actin and membrane surface and condense the cortical actin in a thinner layer (Fig 3A, middle panel and Fig 3F).

**Fig 4:**
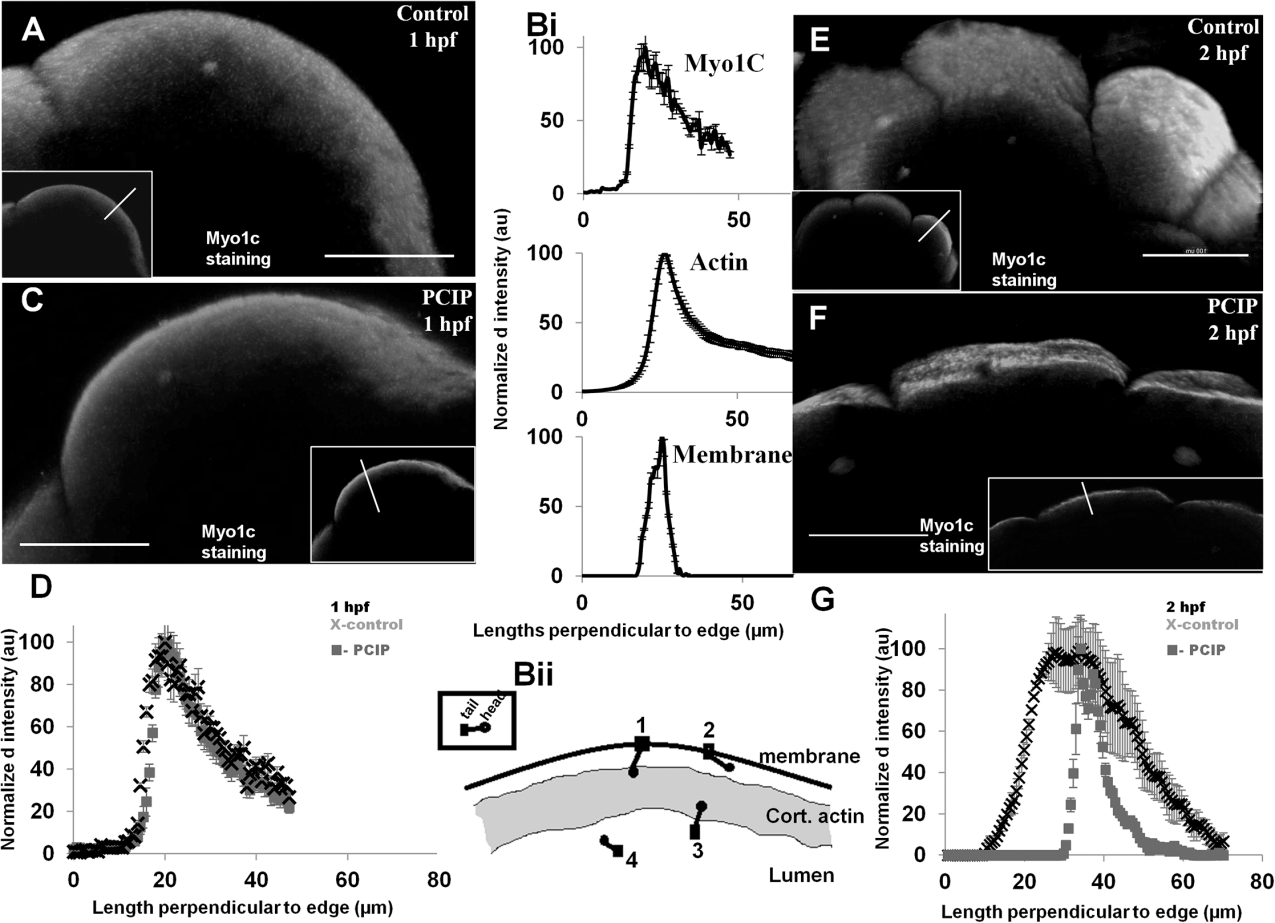
Redistribution of blastomeric Myo1C upon MyoI inhibition by PClP. (A) 3D rendered 100 μm cross section of Myo1C immunostaining profile in control 1 hpf embryo (inset- single confocal slice) (Bi) Normalized immunostaining intensity along the line drawn in Fig 4A inset, n = 5 embryos (Bi, top panel). Actin distribution at the same time, redrawn from Fig 3A, left panel, n = 5 (Bi, middle panel). Membrane distribution profile perpendicular to surface of embryo shown in Fig 3D, control)\, (Bi, bottom panel). (Bii) cartoon representation of Myo1C distribution in blastodisc cortex. (C) 3D rendered in 100 μm cross section of Myo1C immunostaining profile in 30 min PClP treated 1 hpf embryo, inset single confocal slice. (D) Comparative normalized intensity calculated long lines drawn in insets of (A&C)-slice views, control-cross, Myo1 inhibited-box, n = 5,. (E) Myo1C profiles for control 2 hpf, (F) Myo1 inhibited 2 hpf and (G) Normalized immunostaining intensity long lines drawn in insets of (E&F)-slice views, control (cross), Myo1 inhibited (box), n = 5, error bar indicates -standard deviation everywhere in Fig 4.

We used immuno-staining to investigate changes in Myo1C distribution post PClP treatment [22] (Fig 4). The antibody used is specific for Myo1C and free from artifacts caused by purely secondary antibody staining (S4 Fig). Myo1C was chosen as the candidate as it can resist a wider range of load in the attached membrane at the C-terminus than Myo1B [44]. Since blastulation in Zebrafish causes large-scale and rapid morphological changes in the cell, we believe this could lead to wider range of cortical tension. This hypothesis is supported by the observations that in Zebrafish embryo, cell-cortex tension varies widely between <50 to >100 μN/m in germ layer progenitor cells [45].

We observed that Myo1C distribution in ~1 hpf control embryo was cortical, and similar to actin distribution (Fig 4A, Myo1C distribution was measured along the line in Fig 4A inset and compared with that of actin in Fig 3A). Actin and Myo1C had equivalent thickness (Fig 4Bi top panel, FWHM Myo1C 14 ±3μm and Fig 4Bi middle panel, FWHM actin 13.26±2.45 μm, actin data from Fig 3C). At 1 hpf, cortical actin and Myo1C distribution were wider than observed for membrane scaffold (FWHM membrane, 7±0.5 μm), measured from embryos stained with membrane dye (Fig 3D & Fig 4Bi bottom panel). Vertebrate Myo1 family proteins have soluble and membrane bound fractions [46]. For Myo1C, a 1:10 ratio for cytosolic and membrane fraction has been reported [47]. We can therefore define four different pools of Myo1, bound to (1^st^) actin and membrane both, (2^nd^) membrane only, (3^rd^) actin only and (4^th^) neither membrane nor actin (Fig 4Bii). The 4^th^pool of Myo1 is very small compared to other fractions, because Myo1C staining was found largely in the cortical region (Fig 4A). We noticed a wide distribution of Myo1C staining away from the membrane and overlapping the actin cortex region, suggesting existence of pool three (Fig 4Bi). The literature suggests, binding to membrane increases the affinity of Myo1C for actin by a factor of 10 [4]. Therefore, we believe that most Myo1C bound to membrane also binds to actin, indicating the existence of significant amounts of 1^st^pool and very low amounts of 2^nd^pool. Therefore 1^st^pool and 3^rd^could be the most prevalent state of Myo1C in cortex. We tested the re-distribution of pool one and three Myo1C on PClP addition. PClP immediately decreased the affinity of Myo1 for actin by >30 fold [15]. We therefore reasoned that addition of PClP converts 1^st^pool and 3^rd^ into 2^nd^pool and 4^th^ respectively. Loss of Myo1 from actin cortex has a significant effect on its structure [42]. As a result, we observed a quick change in the actin cortex thickness upon PClP addition (Figs 3A and 4B, middle panels). However, concurrent change in Myo1C profile was not observed at 1 hpf (Fig 4A, 4C and 4D). Since Myo1C is shown to interact with endoplasmic reticulum, extensive ER structure which is present in cortex might have temporarily slowed down the diffusion of freed Myo1 pool four [42, 48, 49]. F-actin network that support ER is also dependent on Myo1C for stability [42]. Therefore, we hypothesize that those ER structure might get destabilized in PClP treated embryo by 2hpf, due to loss of supporting actin structure. Since the affinity of Myo1C for membrane binding remains unaffected upon PClP treatment, the blastodisc membranes could act like a trap for Myo1C. Presence of such a trap could hold a fraction of Myo1C from escaping. In agreement with this hypothesis, we found that at 2hpf, only a thin layer of Myo1C bound to membrane was left (Fig 4F and 4G). At the same time, we found a wider distribution of Myo1C in control (Fig 4E–4G). At present, it is not clear whether in the 2 hpf PClP treated embryo the fraction of Myo1 which remains bound to membrane is also bound to cortical actin.

Taken together, we conclude that changes in the Myo1 distribution, along-with apparent changes in membrane cytoskeletal adhesion might have changed the cortical actin structure as reported above (Fig 3). 3D architecture of membrane follows cortical actin organization, as it is attached to the actin-cytoskeleton by other membrane-cytoskeleton adhesion proteins like ERM, Band3-proteins, spectrin even in absence of a functional Myo1 [50]. This raises a possibility that any change in cortical region is simultaneously felt by actin and membrane and might results in wrinkles in membrane surface on treatment with PClP (Fig 3D and 3H). Taken together, our experiments have shown that the action of PClP on early Zebrafish embryo leads to changes in blastodisc surface and distribution of cortical actin and Myo1C. Further biophysical experiments can be planned to see if this change is associated with changes in the membrane tension, due to abolition of motor activity of Myo1 in PClP treated embryos.

### Myo1 is critical for maintaining the distribution of LDs in blastodisc

We observed that during cell division, cortical LDs exhibit a dynamic movement around cleavage furrow and their motion got affected differently upon PClP and Blebbistatin treatment (Fig 2A and 2B, S1 and S2 Movies). Since LD motion is dependent on f-actin, we compared LD motion around first cleavage furrow in details in the presence and absence of Myo1 -and Myo2- inhibition.

Various enzymes which synthesize phospholipids, reside on LDs [51]. Therefore, we speculated that LDs could be a source of lipids to the newly forming furrow during cell division. However, other mechanisms like exocytosis, also exists and could supply lipids to the newly forming furrows [52–54]. In PClP treated embryos, furrows which have matured beforehand, might not require excess lipid. Contrary to this, we observed gradual LD accumulation near the first furrow (Fig 2B bottom panel). The resulting accumulated LD clump was apparently equal or more prominent in first furrow than third furrow (S1 Movie). Whereas, in control embryos, we see a transient accumulation of LDs near the furrows during their formations (Fig 2A and 2B top panels, S1 and S2 Movies). Therefore, we hypothesize that, Myo1 is critical in maintaining the distribution of LDs in the blastodisc. To investigate this, we analysed first cleavage furrow region in higher magnification (lateral view), since this region has higher concentration of LDs (Fig 2B).

**Fig 5:**
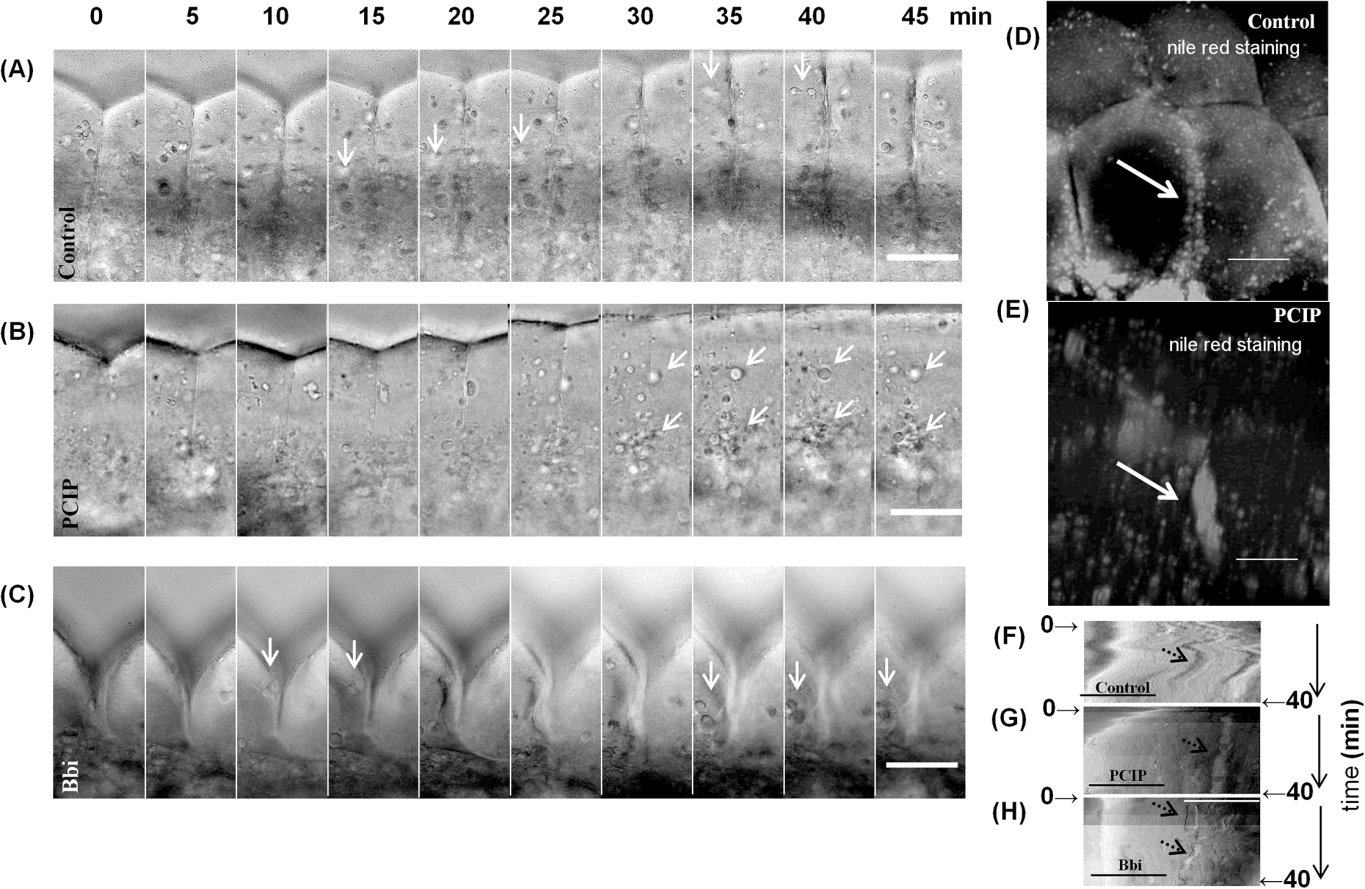
LDs gradually accumulate at the cleavage furrow of PClP treated embryo. (A-C) Lateral views of embryo, as observed in Fig 2A, Zoomed in on first cleavage furrow, (A) Control embryo from approximately 40 min post fertilization, montage of every 5 min, arrows indicates LDs near the first cleavage furrow. (B) Myo1 inhibited embryo from approximately 40 min post fertilization, montage of every 5 min. Arrows indicate accumulation of LDs at the first cleavage furrow line. (C) Myo2 inhibited embryo from approximately 40 min post fertilization, montage of every 5 min. Arrows indicate LDs near the first cleavage furrow. (D) Nile red staining of first cleavage furrow with lipid droplets shown by arrow in control embryo. (E) Nile red staining of first cleavage furrow with lipid droplet clump shown by arrow in Myo1 inhibited embryo. (F) Control, (G) Myo1 inhibited and (H) Myo2 inhibited embryos, kymograph view of average intensity along 10 μm line on both sides of the first cleavage furrow, ~ for approximately 40 min as shown in panels 5A-5C. Time is indicated along vertical axis and correlates with images in panel (5A-5C), approximate location of start and finish of 40 min time in kymographs are marked by arrows. LDs are indicated by black arrowheads. Scale bars are 50 μm in all images of this figure.

In control and blebbistatin treated embryos, the LDs exibit in and out movement with respect to the first cleavage furrow (arrows, Fig 5A and 5C, S4 Movie). This movement of LDs is associated with its active and inactive states [11]. In case of Myo1 inhibited embryos, we did not observe such in and out motion of LDs. Rather, the LDs accumulated at the furrow, until the furrow could no longer be detected (Fig 5B arrows, S4 Movie). We verified that the accumulated materials at the furrow were LDs by nile red staining (Fig 5D and 5E) [55]. Unlike hollow vesicle staining, we observed that in PClP-treated embryos, the accumulated clumps on the furrow stained strongly with nile red as filled spheroids, suggesting that the clumps are primarily LDs (Fig 5E arrow, panels C-D in S5 Fig). In control embryos, we found LD distribution of either side of the furrow and no clump could be seen (Fig 5D, panels A-B in S5 Fig). Taken together, we have shown that there was unique lipid-clump formation due to Myo1 inhibition. We further studied the clumps by kymograph and tracking of LDs in bright field images.

To demonstrate the differences in temporal dynamics of LD, we generated kymographs for LD- movement corresponding to control, Myo1 inhibited and Myo2 inhibited embryos (Fig 5F–5H). We measured the average intensity (in bright field) of a region 10 μm on either side of the furrow and plotted them for the entire length of furrow in horizontal axis, with time in the vertical axis. In control and blebbistatin-treated embryos, periodic appearance and disappearance of LD traces could be seen, suggesting transient recruitment of LDs to the furrow (arrows, Fig 5F and 5H). However, LDs in PClP treated embryos did not move away from the furrow and stayed connected, leading to clump formation (arrow, Fig 5G).

To further analyze the detailed LD accumulation in first cleavage furrow, we tracked LDs by MTracJ plugin of ImageJ [56]. Unlike control, LD tracks of PClP treated embryos showed biased motion towards cleavage furrow and loss of active and inactive phase (S5 and S6 Movies). The differences in biophysical nature of LD movement between control and PCIP treated embryos are discussed in details in supplementary section (see S1 Text).

Taken together, we conclude that inhibiting Myo1 leads to the accumulation of LDs near the cleavage furrow, upsetting its distribution.

### Critical roles of Myo1 in early embryogenesis

We have summarized the phenotypes associated with the inhibition of Myo1 activity by PClP in early Zebrafish embryo. In addition to being involved in membrane cytoskeletal adhesion and transport, Myo1 plays a critical role in early embryonic development. Egg-cells are huge compared to somatic cells. In case of mammals, where there is less dependency on yolk and placenta provides the nutrients, eggs have diameter of 100μm, compared to 10–12μm in somatic cells [57]. These large cells may need Myo1 for mechanical stabilization and regulation of thick cortical actin and associated membrane. Myo1 could also bring new membranes during cleavage furrow formation.

Development is a complex phenomenon that is regulated through the action of a number of molecules. Effect of Myo1 inhibition by PClP is reversible as shown in cell culture-based experiments [16]. However, till date, we could not detect reversibility in relatively complex Zebrafish embryogenesis. Therefore, our lead data on Myo1 function in development need to be explored in further details, to obtain more mechanistic insights. We believe that our data on role of Myo1 in early development are well supported and allow drawing logical conclusion. However, one should be cautious, as concentrations of drugs reported *in vitro* and in cell cultures sometimes have different effects in whole Zebrafish embryos [58]. Follow up experiments need to involve co-injection of Morpholinos against several Myo1 isotypes together in mature oocytes, thus allowing sufficient time to delete all those proteins in early (1–8 cell) embryos [59, 60]. Simultaneous knockdown against multiple Myo1s is critical as they have overlapping functions [4]. Such morpholino-cocktail composition needs to be determined carefully, as inhibition of all Myo1s in cells (by PClP) shows drastic phenotype [16]. In oocytes, action of morpholino-cocktail may lead to failure of fertilization. Such an endeavor is challenging and may require a significant amount of preliminary work. Therefore, we consider that is beyond the scope of this paper. Importantly, we have laid the foundation of work that shows Myo1 molecules are important for the development of early embryos.

## Materials and methods

### Zebrafish culture, breeding and drug treatment

Zebrafish culture was performed in compliance with Indian Association for the Cultivation of Science Animal Ethics Committee and good laboratory practice developed in house [11]. All Zebrafish handling and breeding techniques wereused in identical ways as done in recent published work [11]. All live embryos were collected and maintained in E3 media (50 mM NaCl, 0.17 mM KCl, 0.33 mM CaCl2, 0.33 mM MgSO4) for all experimental purpose. Dechorionation of embryos was carried out by pronase, deyolking was done using published protocol [11, 61]. Blebbistatin and PClP treatment were done in E3. We used 75 Zebrafish eggs for making extracts for each western blot lysate.

### Semi-quantitative rt-PCR of Myo1 isoforms

RNA isolation using a 35 cycle semi-quantitative RT-PCR was carried out following published protocols with minor modifications [62]. The embryos were homogenized by repeated pumping thru’ sterile clinical injection needle instead of a pellet pestle. Following cDNA preparation by oligo-dT primer method using a BioBharti cDNA kit, 34 cycle PCR was carried out by using the following primers.

ActinF: atggatgatgaaattgccgca; ActinR: ctgtgtcatcttttccctgttgg; Myo1EaF: cctgagtcgctattcctgctgg
Myo1EaR: ggtctctcttaatgctcttaaacctcct; Myo1EbF: gacatcttcatattgcacgaggatc
Myo1EbR: cgaccagcagacttaggagcttc; Myo1CbF: tgccaaaggagaagagctgat; Myo1CbR: tgcacaggctcccacgtg; Myo1BF:tgctcttaagcttagagagggtgcta; Myo1BR: tgtcctgcttaaactgtaccaagaactc.

### Western blot and immuno-staining of Myo1C

Western blot for Myo1C and immuo-staining of Zebrafish embryos were carried out by following the standard protocols published for the same antibody [22]. For western blot, lysate from equal number of embryos were compared for up or down regulation of expression, taking β-actin band as control.

### Staining of nucleus, actin, membrane, and lipid droplets

DAPI staining was used for nuclear visualization. Reference of DAPI stained nucleus was used to count the number of cells present in balstomere, wherever cell septa were not visible due to Myo1-inhibition. F-Actin and lipid droplets of fixed Zebrafish embryos were stained following published protocol[11]. For membrane staining, CellMask™ (Thermo-fisher) reagent was used, following manufacturer’s protocol.

### Optical microscopy, image processing and data analysis

Bright Field and confocal microscopy of Zebrafish embryos were carried out using Olympus BX61 or LeicaTCS SP8 microscopes using low magnification 20X objective. Embryos were embedded live in 0.8% low melting agarose ±drugs for imaging purpose. Unless otherwise stated, drugs were added in the embedding media, when the first cleavage furrow was forming. For image processing and 3D rendering of Zebrafish embryos, ImageJ, IMARIS^TM^ and MATLAB softwares were used. Basic calculations and graph plotting were carried out by Microsoft EXCEL ^TM^.

### Scanning electron microscopy (SEM)

SEM of Zebrafish embryos was carried out in in-house FE-SEM microscope. Embryos were fixed in 2.5% glutaraldedye and negative stained by 4% OsO_4_.

### TIRF microscopy

TIRF microscopy was carried out using a 100X TIRF lense (NA = 1.49) in an Olympus TIRF microscope. 4% PFA fixed Embryos were stained with phalloidin-FITC and sandwiched between two glass sildes 700 nm apart. Blastomeres of some of the embryos were found touching the glass surface and TIRF imaging were done for those embryos.

### Data management and statement of statistical analysis

Western blot trials were carried out with embryos from three separate pair of fish in three independent experiments.

For description of shrinkage of blastomere (Fig 1E), 8 sets (10 embryos each in control and test in each set) of individual experiments were carried out. Each experiment had embryos from an independent parent-fish. Average blastomere height of all embryos in each set was considered as a single value (n = 1). Eight such sets of values (n = 8) where plotted in the graph with SD values. Plots have shown high confidence (two tailed p = 0.0024, at 2 hpf).

Whenever thickness of acin/membrane/Myo1C was measured, one embryo each from each set was picked for confocal imaging. As indicated by “n = 5” in figure legend of Figs 3 & 4, 5 embryos from 5 different breedings were compared.

In Fig 3C, the two tailed p = 0.0002, n = 5 between actin cortical thickness of control vs PClP.

In Fig 3C, the two tailed p = 0.0015, n = 5 between actin cortical thickness of Bleb vs PClP.

In Fig 4G, the two tailed p = 0.0011, n = 5 between the thickness of Myo1C layer at FWHM, control vs PClP treated embryos.

### Animal ethics statement

All animal experiments were carried out according to the guidelines approved by the Indian Association for the Cultivation of Science Animal Ethics Committee. Appropriate measures were taken to minimize pain or discomfort to animals.

Our experimental embryos are below 3 hours post fertilization (3hpf) and at this early stage embryos do not start feeding by themselves, they were approximately at 1000 cell stage, if untreated by drugs [35].

Since we are dealing with non-self-feeding early embryos (maximum 3 hpf age) and not the live fish, we are not required to submit any statutory (eg IACUC) clearance [63].

At the end of the fertilization day, all live embryos [still not self feeding <1dpf [35]] are donated back to the fish farmer, who sold us the adult fish or dropped live in the rice paddy [the natural habitat of Zebrafish [35]]. We did not euthanize any fish egg.

## Supporting information

S1 Text Biophysical nature of LD motions, changes in direction and instantaneous speed of LD motion upon PClP treatment. (PDF)

S1 Fig **(A)** Example of a two cell stage embryo, where drug was added. **(B-C)** Difference in cell division arrest phenotype, top-view position for **(B)** control by (**C)** 2.5 μM PClP. **(D-F)** Difference in cell division arrest phenotype, lateral view, extended time for **(D)** control by **(E)** 100μM blebbistatin and **(F)** 2.5 μM PClP. Bar 100 uM.
(PDF)

S2 Fig Time course of complete regression of cleavage furrow when 100 μM blebbistatin was added at the one cell stage (two experiments), top-view. Bar 50 μm.
(PDF)

S3 Fig Cortical actin profile by TIRF of two embryos each, for control, PClP/Myo1 inhibition and blebbistatin treatment/Myo2 inhibition (at 1 hpf/30min treatment for either drug). Actin appears mainly as dense sheet like structure in control and Myo2 inhibited treated embryos, but appeared tubular in Myo1 inhibited embryos (arrows). (bar 5 μm).
(PDF)

S4 Fig Zebrafish embryo, stained with Myo1C primary and secondary antibody showed cortical staining, whereas only secondary antibody showed yolk and background staining. Bar 100 nm. (PDF)

S5 Fig LDs at the first cleavage furrow of (A-B) Control 8 cell, (C) 2 hpf Myo1 inhibited 8 cell-sideview, (D) 2 hpf Myo1 inhibited 8 cell-topview.
(PDF)

S6 Fig (A) Cartoonic representation of sidewise orientated embryo with enclosed region by dashed line represent the region in which LDs were tracked for plots in S6B–S6E Fig. (B, D) LDs’ distance from first furrow, (C, E) LDs’ distance from the yolk blastomere interface, in S5 and S6 Movies. (B, C) control, (D, E) PClP treated. Arrow in D indicate bias towards furrow, Arrows in C&E indicate minor bias away from yolk and towards cortical region.
(PDF)

S7 Fig (A) Average instantaneous speed of LDs in control for 30 min in the dotted region as in S6A Fig, encompassing 3^rd^ furrow formation (B) Average instantaneous speed of LDs in Myo1 inhibited embryo for 30 min in the same region, encompassing 3^rd^ furrow formation. Average of 180 LDs taken from data sets of S5 and S6 Movies, error bars- standard-error. (C) Control (arrows) and (D) Myo1 inhibited (arrows) LD tracks of S5 Movie and A&B panels above, 0–10 mins, vertical lines indicate cleavage furrows, bar 50μm.
(PDF)

S1 Movie Side view imaging of (Top-panel) PClP treated, (Middle-panel) Blebbistatin treated and (bottom-panel) control embryos for 96 min from first cleavage furrow (marked as “1”) formation. Third furrow is marked as “3”. Images were captured every 24 sec and played at 30 fps. Movie data analysed in Fig 2A. Bar 100 μm.
(MOV)

S2 Movie Top view imaging of (Top-panel) control, (Middle-panel) Blebbstatin treated and (bottom-panel) PClP treaed embryos for 51 min from first cleavage furrow formation. Images were captured every 10 sec and played at 30 fps. Movie data analysed in Fig 2B. Bar 50 μm.
(MOV)

S3 Movie Z-sections of cortical actin profile of (Left panel) control, (middle panel) PClP treated and (right panel) blebbistatin treated embryos. Movie data analysed in Fig 3A, Bar 100 μm.
(MOV)

S4 Movie Zoomed-in movie on first cleavage furrow for of (Left panel) control, (middle panel) PClP treated and (right panel) blebbistatin treated embryos for 45 min duration from the formation of first cleavage furrow. Images were captured every 2.7 sec and played at 10 fps. Movie data analysed in Fig 5A–5C. Arrow in Fig 5B/middle panel indicates position of clump formation. “A” and “I” in control panel denotes active and inactive phases of LD motion. Bar 50μm.
(MOV)

S5 Movie First example of comparative LD tracking on either side of the first cleavage furrow in the boxed region as shown in S6A Fig, between (left panels) control and (right panels) PClP treated embryos. Images were captured every 8 sec and played at 24 fps. Insets, full view of the side faced blastomere. Bar-50μm. Movie data were analyzed in S6 & S7 Figs.
(MOV)

S6 Movie Second example of comparative LD tracking on either side of the first cleavage furrow in the boxed region as shown in S6A Fig, between (left panels) control and (right panels) PClP treated embryos. Images were captured every 8 sec and played at 24 fps. Insets, full view of the side faced blastomere. Bar-50μm. Movie data were analyzed in S6 & S7 Figs.
(MOV)
