# Supplementary material for: Myosin-1 inhibition by PClP affects membrane shape, cortical actin distribution and lipid droplet dynamics in early Zebrafish embryos
Source: PLoS One. 2017 Jul 5;12(7):e0180301. doi: 10.1371/journal.pone.0180301 (PMC5498032; doi:10.1371/journal.pone.0180301)
Supplement: S1 Text — (PDF) [file pone.0180301.s001.pdf]

Supplemental material

Supplemental text captions:

S1 text. Biophysical nature of LD motions, changes in direction and instantaneous speed of LD motion upon PClP treatment

We imaged for ~30 min time period between the formation-time of second and third furrow in control and PClP treated embryo, in the 100 μm region on either side of first cleave furrow as indicated in the dotted box (Panel A in S6 Fig). We made two of such sets of movies, one control and one Myo1 inhibited embryo per set (S5 and S6 Movies). We have plotted the absolute distance of droplet from the first cleavage furrow for control and Myo1 inhibited embryos in the dataset shown in the movies (Panels B & D respectively in S6 Fig). We also plotted the absolute distance from the approximate yolk-blastomere interface for the control and PClP treated embryos (Panels C & E respectively in S6 Fig). We observed a noticeable bias of LD movement towards cleavage furrow in Myo1 inhibited embryos (panel D in S6 Fig - dotted arrow, S5 and S6 Movies), however the distance of LDs towards furrow were largely random for control embryos (S6 Fig panel B, dotted arrow, S5 and S6 Movies). For movement w.r.t yolk-blastomere interface, both control and Myo1 inhibited embryos showed a minor bias away from yolk, otherwise their movement appeared random (Panels C&E in S6 Fig- arrows, S5 and S6 Movies). Therefore, we observed a directed motion of the LDs towards the furrow upon Myo1 inhibition, leading to clump formation. What causes the biased motion remains unknown and needs to be explored further.

We compared the averaged instantaneous speed of LDs two independent experiments as above for control and Myo1 embryos, for 30 min (S5 and S6 Movies). In control embryos, we found the well characterized active and inactive phase in movement, as publish before (Panel A in S7 Fig) (Dutta and Kumar Sinha, 2015). The LDs are mobile except when the 3rd furrows were forming (Panel A in S7 Fig). But in the Myo1 inhibited embryos, we could not detect such phased movement and LDs gradually stop moving when the 3rd furrow gradually got dissolved (Panel B in S7 Fig). This result was in agreement with our hypothesis that LDs form static clump in the first furrow line, as the 3rd furrow got dissolved upon PClP treatment (Fig 5, B, E, G; panels C-D in S5 Fig). A closer look into the S5 and S6 Movies revealed that there was a streaming motion of LDs towards first cleavage furrow in Myo1 inhibited embryos but not in

control embryos where LD motion appeared to be randomly distributed (Panels D and C in S7 Fig). The streaming motion towards the cleavage furrow might be the reason behind clump formation on the first cleavage furrow line.
