# Supplementary figures and images for: Myosin-1 inhibition by PClP affects membrane shape, cortical actin distribution and lipid droplet dynamics in early Zebrafish embryos
Source: PLoS One. 2017 Jul 5;12(7):e0180301. doi: 10.1371/journal.pone.0180301 (PMC5498032; doi:10.1371/journal.pone.0180301)

S1 Fig

A

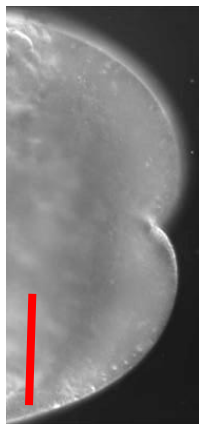

B

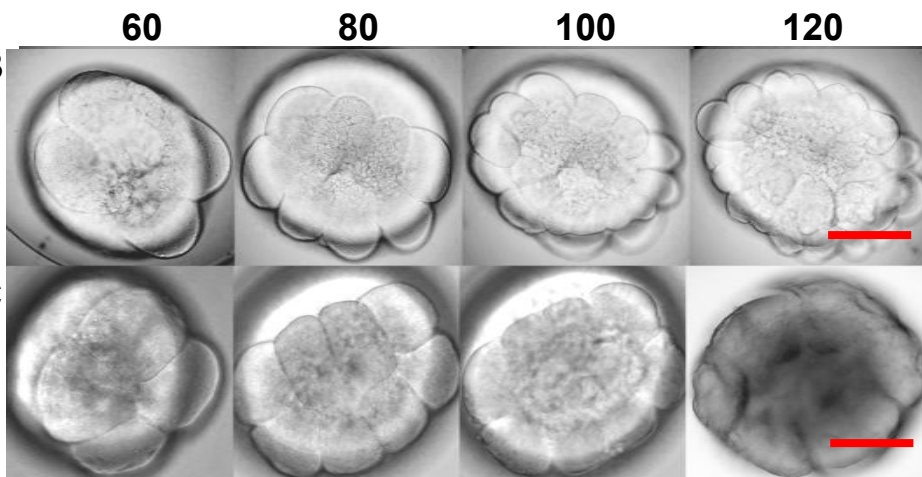

C

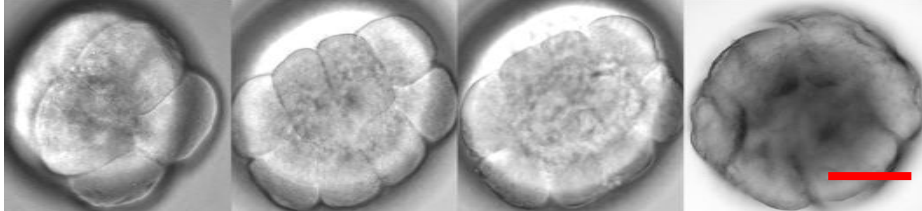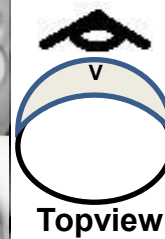

Topview

D

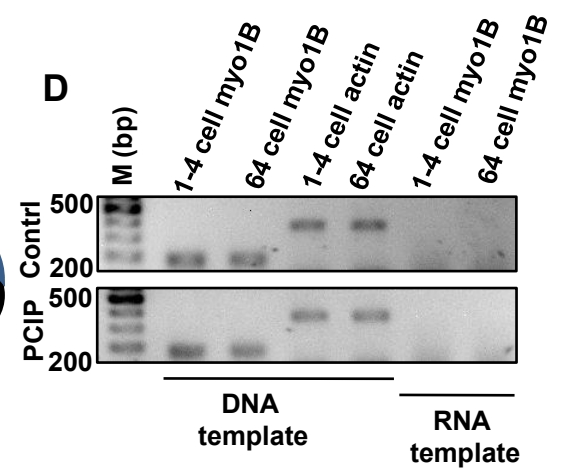

E

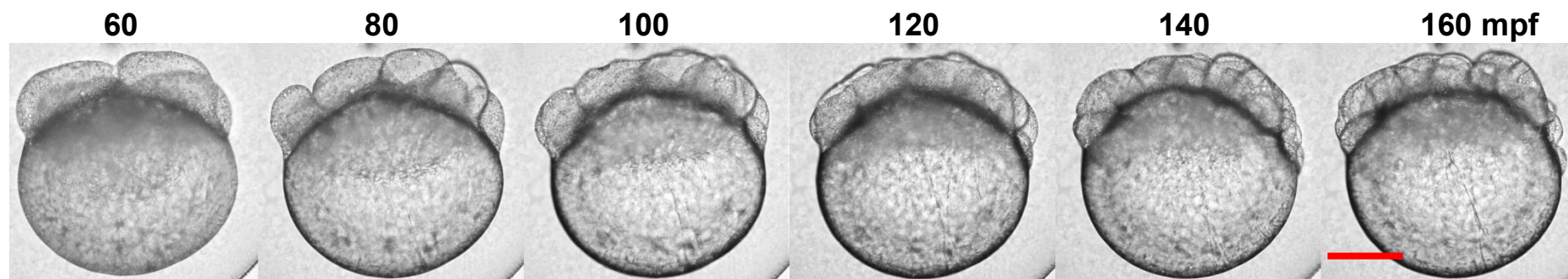

F

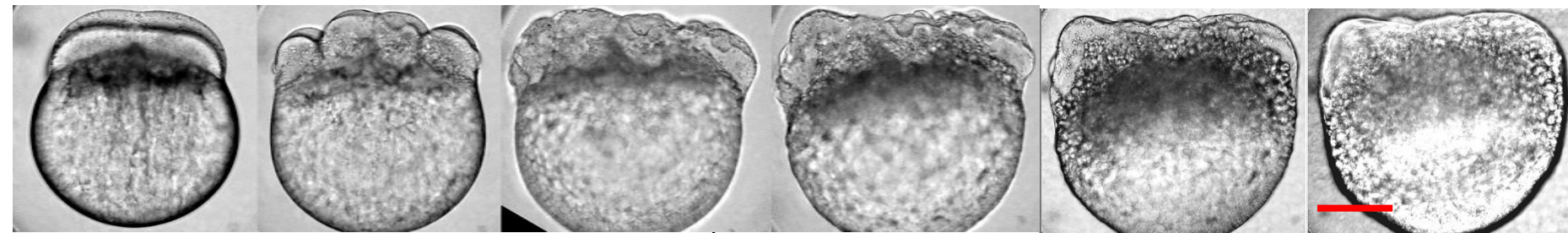

G

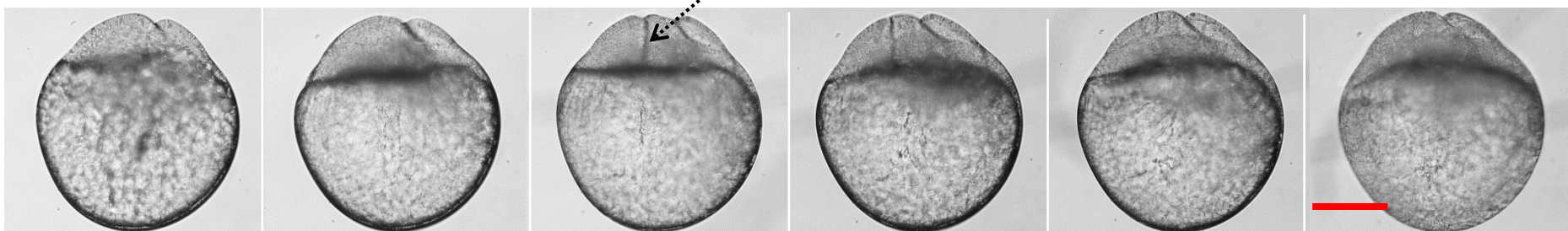

Supplement: S1 Fig — (A) Example of a two cell stage embryo, where drug was added. (B-C) Difference in cell division arrest phenotype, top-view position for (B) control by (C) 2.5 μM PClP. (D-F) Difference in cell division arrest phenotype, lateral view, extended time for (D) control by (E) 100μM blebbistatin and (F) 2.5 μM PClP. Bar 100 uM. (PDF) [file pone.0180301.s002.pdf]

Fig S2

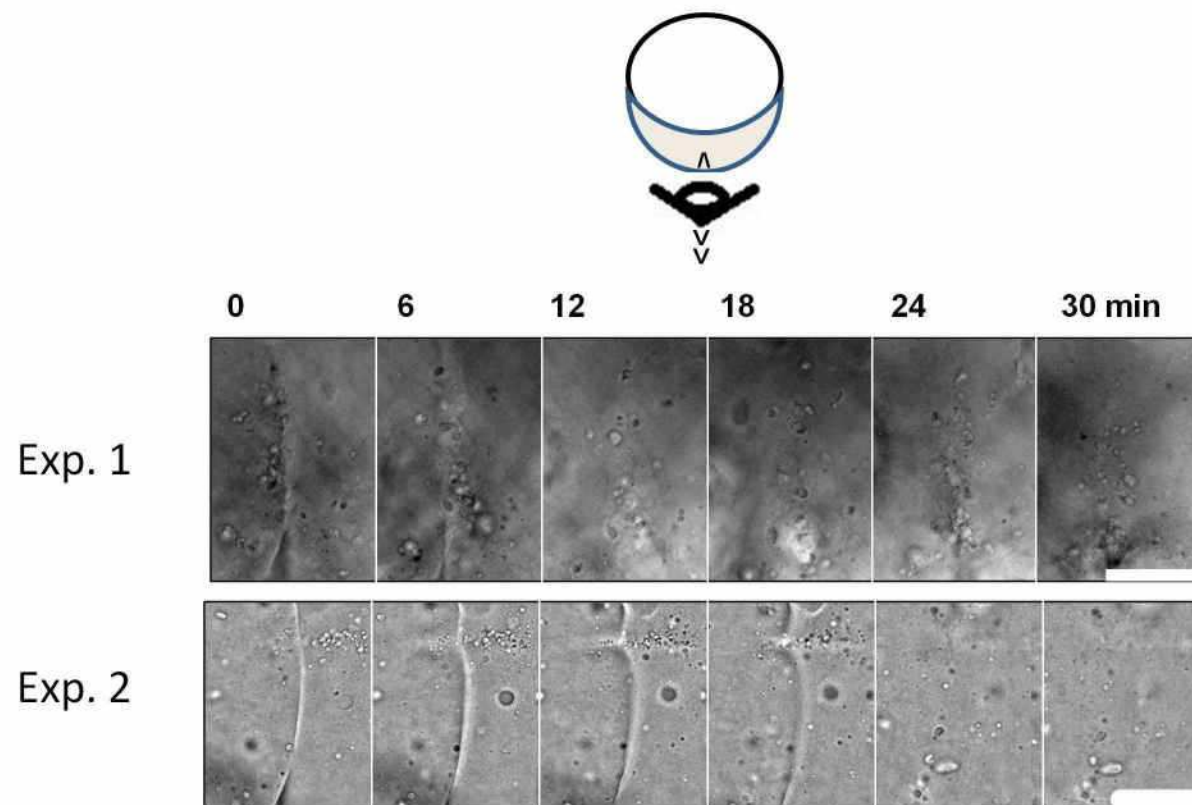

Supplement: S2 Fig — Bar 50 μm. (PDF) [file pone.0180301.s003.pdf]

**Fig S3**

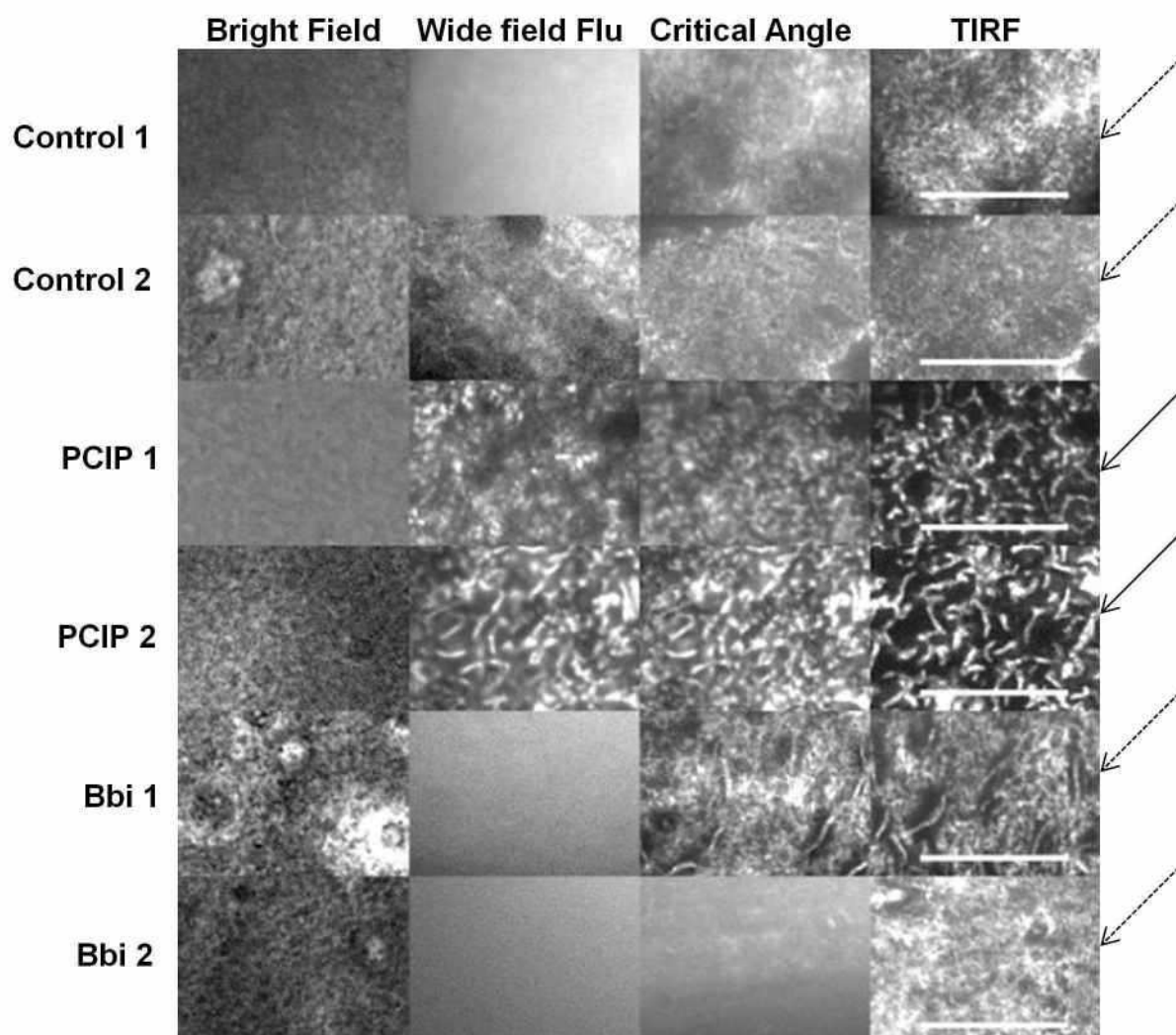

Supplement: S3 Fig — Actin appears mainly as dense sheet like structure in control and Myo2 inhibited treated embryos, but appeared tubular in Myo1 inhibited embryos (arrows). (bar 5 μm). (PDF) [file pone.0180301.s004.pdf]

**Fig S4**

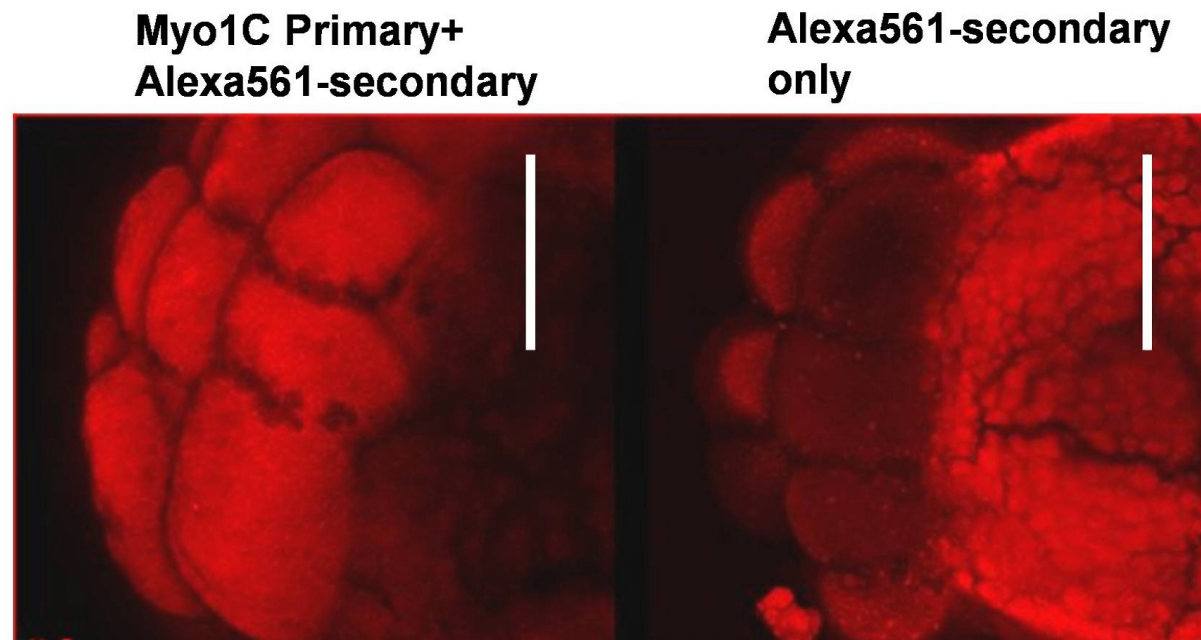

Supplement: S4 Fig — (PDF) [file pone.0180301.s005.pdf]

Fig S5

A

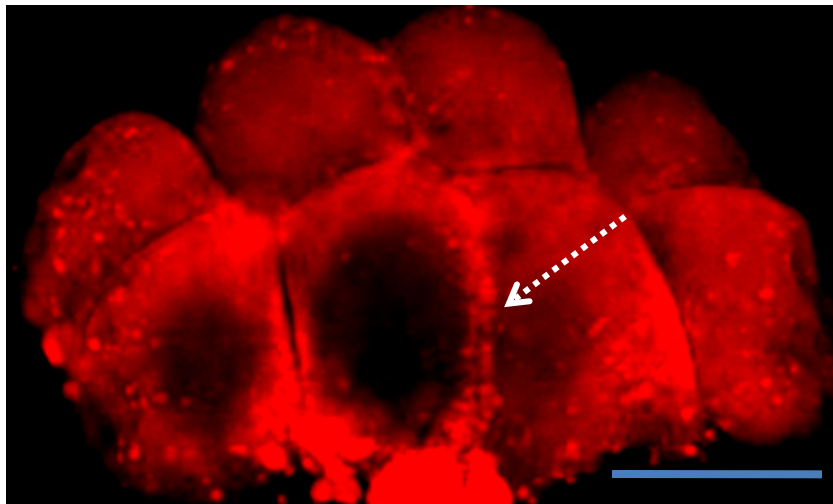

B

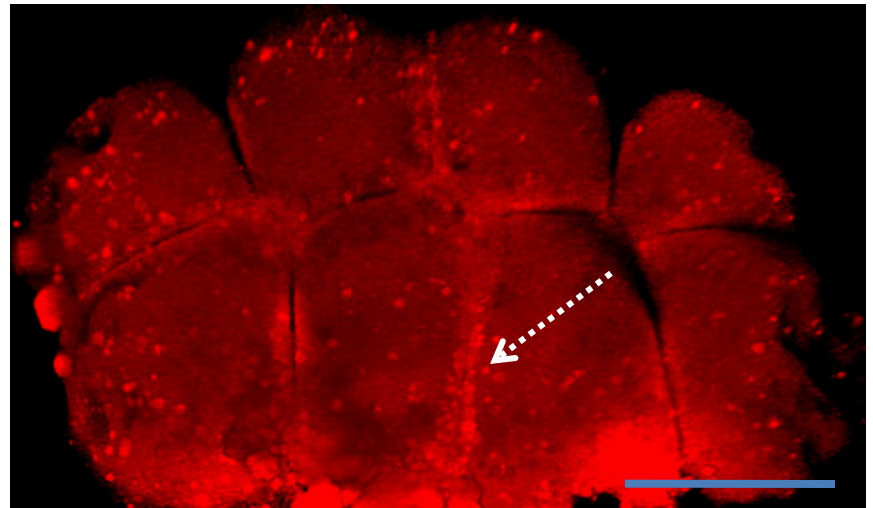

C

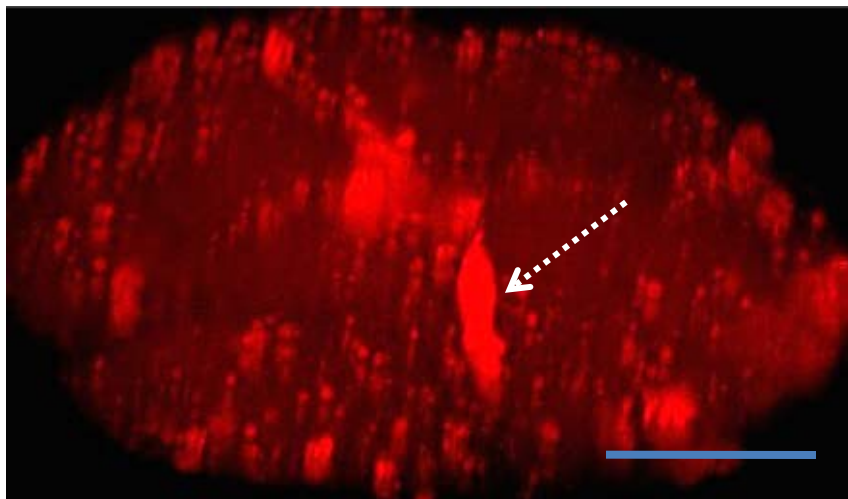

D

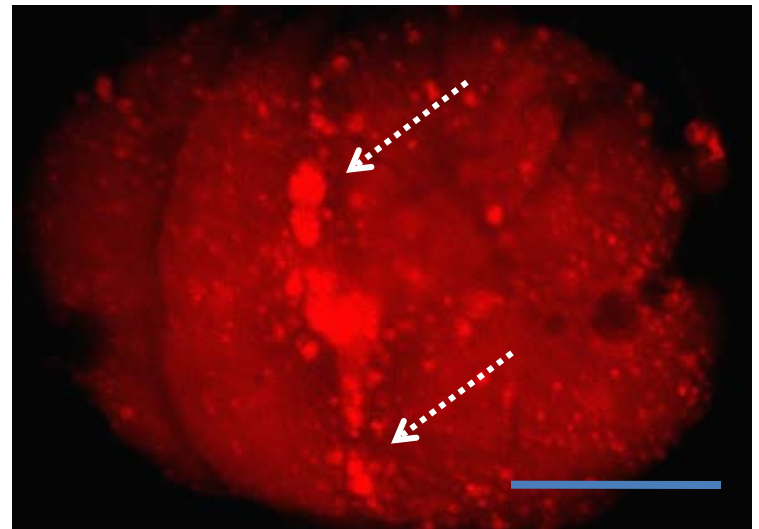

Supplement: S5 Fig — LDs at the first cleavage furrow of (A-B) Control 8 cell, (C) 2 hpf Myo1 inhibited 8 cell-sideview, (D) 2 hpf Myo1 inhibited 8 cell-topview. (PDF) [file pone.0180301.s006.pdf]

Fig S6

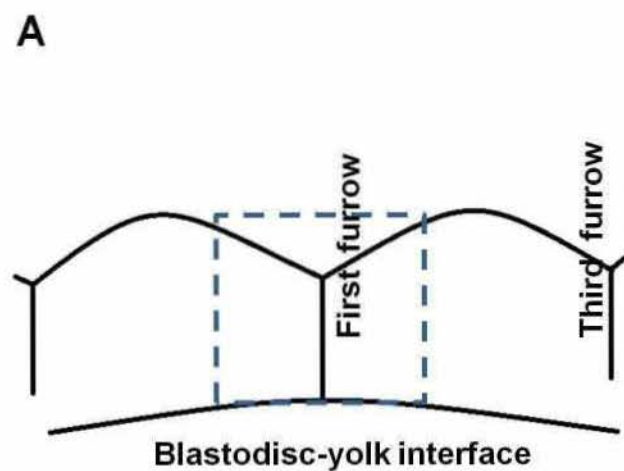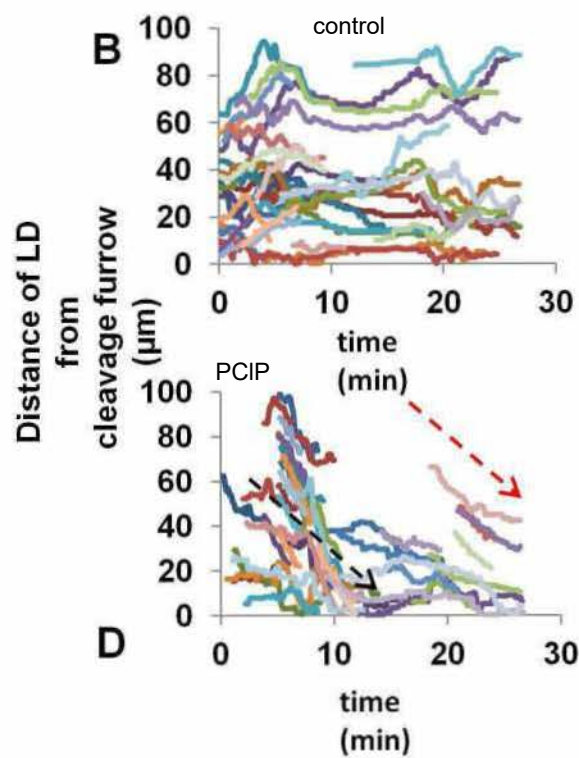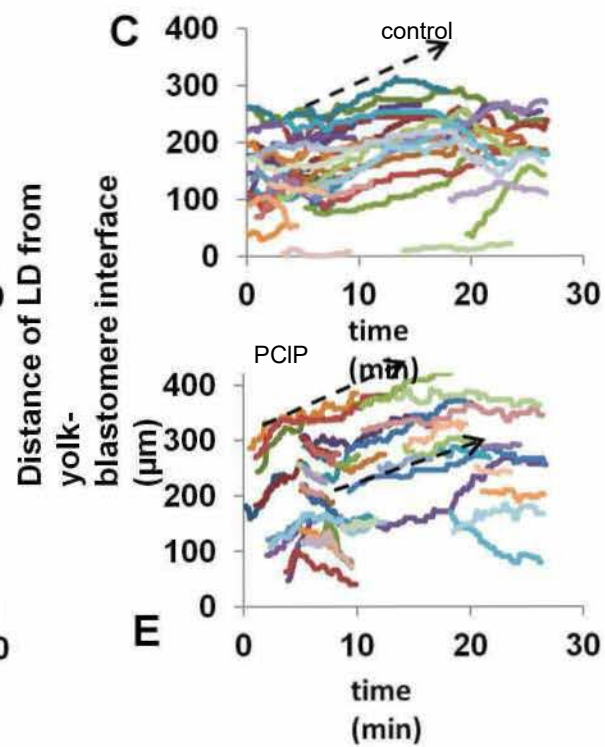

Supplement: S6 Fig — (A) Cartoonic representation of sidewise orientated embryo with enclosed region by dashed line represent the region in which LDs were tracked for plots in S6B–S6E Fig. (B, D) LDs’ distance from first furrow, (C, E) LDs’ distance from the yolk blastomere interface, in S5 and S6 Movies. (B, C) control, (D, E) PClP treated. Arrow in D indicate bias towards furrow, Arrows in C&E indicate minor bias away from yolk and towards cortical region. (PDF) [file pone.0180301.s007.pdf]

Fig S7

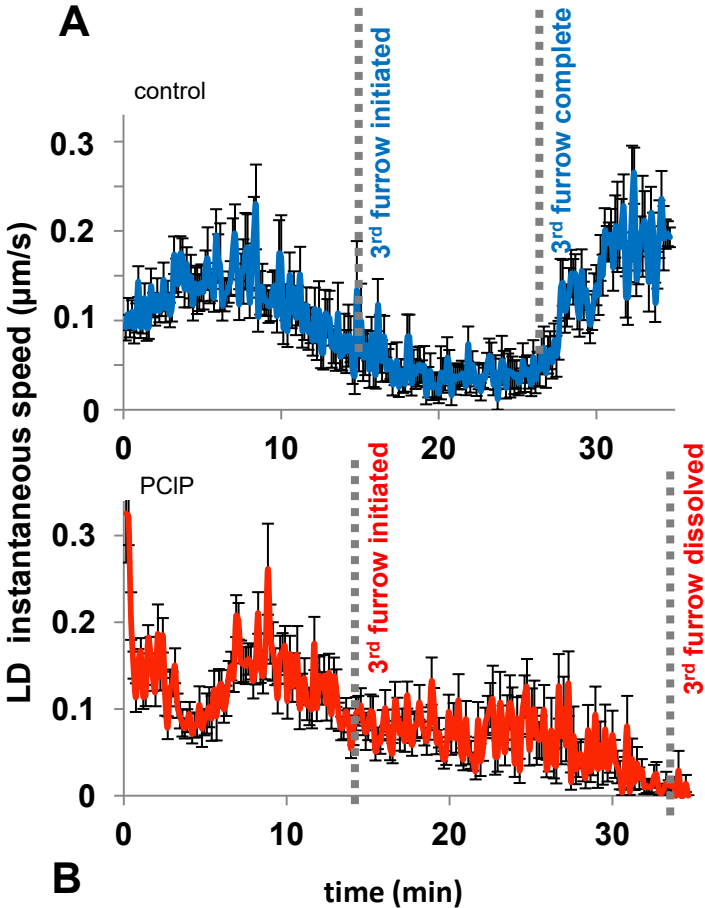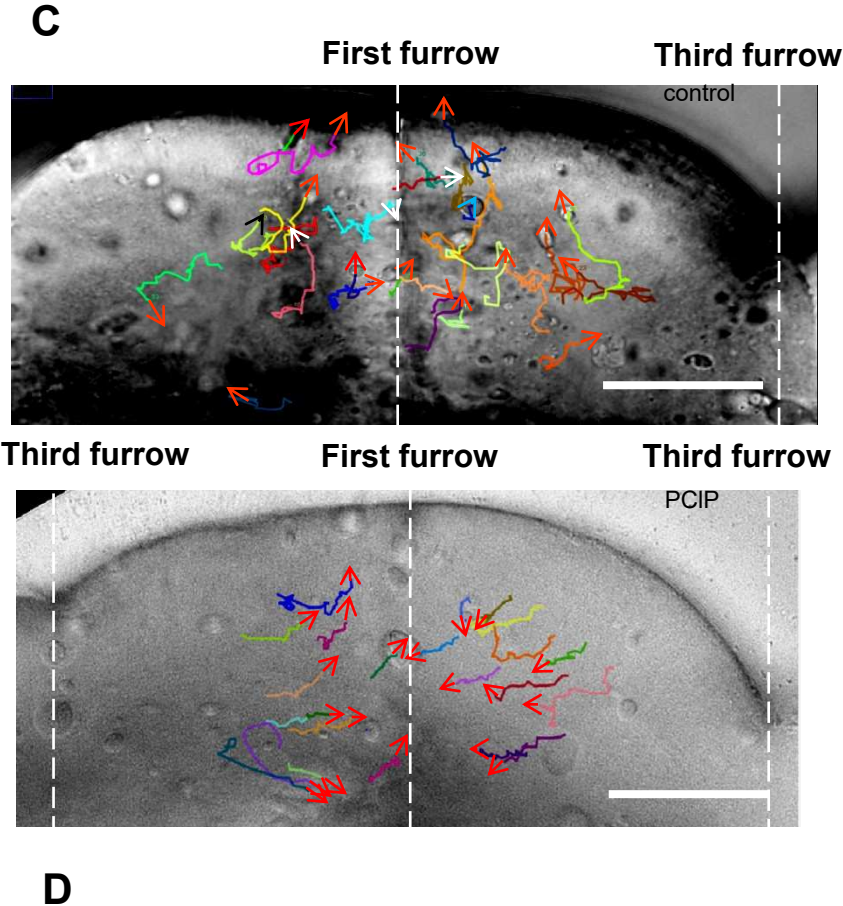

Supplement: S7 Fig — (A) Average instantaneous speed of LDs in control for 30 min in the dotted region as in S6A Fig, encompassing 3rd furrow formation (B) Average instantaneous speed of LDs in Myo1 inhibited embryo for 30 min in the same region, encompassing 3rd furrow formation. Average of 180 LDs taken from data sets of S5 and S6 Movies, error bars- standard-error. (C) Control (arrows) and (D) Myo1 inhibited (arrows) LD tracks of S5 Movie and A&B panels above, 0–10 mins, vertical lines indicate cleavage furrows, bar 50μm. (PDF) [file pone.0180301.s008.pdf]
